# Antibacterial Activities of Selected Cameroonian Plants and Their Synergistic Effects with Antibiotics against Bacteria Expressing MDR Phenotypes

**DOI:** 10.1155/2012/623723

**Published:** 2012-02-28

**Authors:** Stephen T. Lacmata, Victor Kuete, Jean P. Dzoyem, Simplice B. Tankeo, Gerald Ngo Teke, Jules R. Kuiate, Jean-Marie Pages

**Affiliations:** ^1^Department of Biochemistry, Faculty of Science, University of Dschang, Dschang, Cameroon; ^2^Transporteurs Membranaires, Chimioresistance et Drug Design, UMR-MD1, IFR 88, Université de la Méditerranée, Aix-Marseille II, Marseille, France

## Abstract

The present work was designed to assess the antibacterial properties of the methanol extracts of some Cameroonian medicinal plants and the effect of their associations with currently used antibiotics on multidrug resistant (MDR) Gram-negative bacteria overexpressing active efflux pumps. The antibacterial activities of twelve methanol extracts of medicinal plants were evaluated using broth microdilution. The results of this test showed that three extracts *Garcinia lucida* with the minimal inhibitory concentrations (MIC) varying from 128 to 512 *μ*g/mL, *Garcinia kola* (MIC of 256 to 1024 *μ*g/mL), and *Picralima nitida* (MIC of 128 to 1024 *μ*g/mL) were active on all the twenty-nine studied bacteria including MDR phenotypes. The association of phenylalanine arginine *β*-naphthylamide (PA*β*N or efflux pumps inhibitor) to different extracts did not modify their activities. At the concentration of MIC/2 and MIC/5, the extracts of *P. nitida* and *G. kola* improved the antibacterial activities of some commonly used antibiotics suggesting their synergistic effects with the tested antibiotics. The results of this study suggest that the tested plant extracts and mostly those from *P. nitida*, *G. lucida* and *G. kola* could be used alone or in association with common antibiotics in the fight of bacterial infections involving MDR strains.

## 1. Introduction

Bacterial infections are responsible for 90% of infections found in health care services. The emergence of MDR bacterial strains appears as the major cause of treatment failure [1]. Among the known mechanisms of resistances, active efflux *via* resistance-nodulation-cell division (RND) pumps is one of the most occurring system in Gram-negative bacterial strains [2]. Efflux pumps are transport proteins involved in the extrusion of toxic substrates (including virtually all classes of clinically relevant antibiotics). The present work was therefore designed to investigate the antibacterial potential against MDR bacteria expressing active efflux though RND pumps. Medicinal plants of Cameroon used in this study include the fruits of *Citrus medica *L. (Rutaceae), the bulbs of *Allium sativum *L. (Liliaceae) and *Allium cepa *L. (Liliaceae), the seeds of *Carica papaya *Linn (Caricaceae), *Cola acuminata *(P. Beauv.) Schott and Endl. (Sterculiaceae), *Buchholzia coriacea *Engl. (Capparidaceae), *Garcinia kola *Heckel (Guttifeare), and *Garcinia lucida *Vesque (Guttifeare), the seeds and fruits of *Picralima nitida*; the potential of the extract from the above plant extracts to increase the activity of some antibiotics on MDR bacteria was also investigated as well as the role of bacterial efflux pumps in the resistance to the tested plant extracts.

## 2. Material and Methods

### 2.1. Plant Materials and Extraction

The nine edible plants used in this work were purchased from Dschang local market, west region of Cameroon in January 2010. The collected vegetal material were the fruits of *Citrus medica*, the bulbs of *Allium sativum* and *Allium cepa*, the seeds of *Carica papaya*, *Cola acuminata*, *Buchholzia coriacea*, *Garcinia kola*, and *Garcinia lucida*, the seeds and fruits of *Picralima nitida*. The plants were identified by Mr. Tadjouteu Fulbert (Botanist) of the National Herbarium (Yaoundé, Cameroon) where voucher specimens were deposited under a reference number (Table 1). 

The fresh or powdered air-dried sample (1 kg) from each plant was extracted with methanol (MeOH) for 48 h at room temperature. The extract was then concentrated under reduced pressure to give a residue that constituted the crude extract. They were then kept under 4°C until further use.

### 2.2. Preliminary Phytochemical Investigations

The presence of major secondary metabolite classes, namely, alkaloids, flavonoids, phenols, saponins, tannins, anthocyanins, anthraquinones, sterol, and triterpenes was determined using common phytochemical methods as described by Harborne [3]. 

### 2.3. Chemicals for Antimicrobial Assays

Ciprofloxacin (CIP), chloramphenicol (CHL), streptomycin (STR), tetracycline (TET), norfloxacin (NFX), cloxacillin (CLX), ampicillin (AMP), erythromycin (ERY), kanamycin (KAN), and cefepim (CEF) (Sigma-Aldrich, St Quentin Fallavier, France) were used as reference antibiotics. *p*-Iodonitrotetrazolium chloride (INT) and phenylalanine arginine *β*-naphthylamide (PA*β*N) were used as microbial growth indicator and efflux pumps inhibitor (EPI), respectively.

### 2.4. Bacterial Strains and Culture Media

The studied microorganisms include references (from the American Type Culture Collection) and clinical (Laboratory collection) strains of *Escherichia coli, Enterobacter aerogenes, Providencia stuartii, Pseudomonas aeruginosa, Klebsiella pneumonia, *and* Enterobacter cloacae *(Table 2). They were maintained on agar slant at 4°C and subcultured on a fresh appropriate agar plates 24 hrs prior to any antimicrobial test. Mueller Hinton Agar was used for the activation of bacteria. The Mueller Hinton Broth (MHB) was used for the MIC determinations.

### 2.5. Bacterial Susceptibility Determinations

The respective MICs of samples on the studied bacteria were determined by using rapid INT colorimetric assay [4]. Briefly, the test samples were first dissolved in DMSO/MHB. The solution obtained was then added to MHB, and serially diluted twofold (in a 96-well microplate). One hundred microlitres (100 *μ*L) of inoculum (1.5 × 10^6^ CFU/mL) prepared in MHB was then added. The plates were covered with a sterile plate sealer, then agitated to mix the contents of the wells using a shaker and incubated at 37°C for 18 hrs. The final concentration of DMSO was lower than 2.5% and does not affect the microbial growth. Wells containing MHB, 100 *μ*L of inoculums, and DMSO at a final concentration of 2.5% served as a negative control. Ciprofloxacin was used as reference antibiotic. The MICs of samples were detected after 18 hrs of incubation at 37°C, following addition (40 *μ*L) of 0.2 mg/mL INT and incubation at 37°C for 30 minutes [5]. Viable bacteria reduced the yellow dye to pink. MIC was defined as the lowest sample concentration that prevented this change and exhibited complete inhibition of microbial growth.

Samples were tested alone and then, in the presence of PA*β*N at 30 *μ*g/mL final concentration. Two of the best extracts, those from seeds of *Garcinia kola *and* Picralima nitida *fruits were also tested in association with antibiotics at MIC/2 and MIC/5. These concentrations were selected following a preliminary assay on one of the tested MDR bacteria, *P. aeruginosa *PA124 (see Supplemental Material S1 available online at doi:10.1155/2012/623723.). All assays were performed in triplicate and repeated thrice. Fractional inhibitory concentration (FIC) was calculated as the ratio of MIC_Antibiotic  in  combination_/MIC_Antibiotic  alone_ and the interpretation made as follows: synergistic (FIC ≤ 0.5), indifferent (0.5 < FIC < 4), or antagonistic (FIC ≥ 4) [6]. (The FIC values are available in Supplemental Material S2).

## 3. Results

### 3.1. Phytochemical Composition of the Plant Extracts

The results of qualitative analysis showed that each plant contains various phytochemicals compounds such as alkaloids, anthocyanins, anthraquinons, flavonoids, phenols, saponins, tannins, and triterpenes as shown in Table 3. 

### 3.2. Antibacterial Activity of the Plant Extracts

Extracts were tested for their antibacterial activities alone and in combination with PA*β*N on a panel of Gram-negative bacteria by the microdilution method. Results summarized in Table 4 showed that the most active extracts were those from *Garcinia lucida* (MIC ranged from 128 to 512 *μ*g/mL), *Garcinia kola* (MIC from 128 to 1024 *μ*g/mL), and the fruits of *Picralima nitida* (MIC from 256 to 1024 *μ*g/mL). The antibacterial activities of these plant species were recorded against all the 29 studied microorganisms. Other extracts exhibited weak activities against a limited number of strains studied.

### 3.3. Role of Efflux Pumps in Susceptibility of Gram-Negative Bacteria to the Tested Plants Extracts

The various strains and MDR isolates were also tested for their susceptibility to the plants extracts, and reference antibiotic (ciprofloxacin) in the presence of PA*β*N, an EPI. Preliminary tests showed that PA*β*N did not have any antibacterial activity at 30 *μ*g/mL. The association of the PA*β*N with the extracts reduced the MIC values of some of the extracts on some tested bacteria (Table 4). However, most of the studied extracts are not the substrates of the active efflux pumps.

### 3.4. Effects of the Association of Some Plants Extracts with Antibiotics

The strain* P. aeruginosa* PA124 was used to find the appropriate subinhibitory concentration of the antibiotic-crude extract to be tested on other bacteria strains. The association of the extracts of* P. nitida *and *G. kola* reduced the MIC of ten antibiotics (CLX, AMP, ERY, KAN, CHL, TET, FEP, STR, CIP, and NOR) at MIC/2 and/or MIC/5 explaining the use of such concentrations. The associations of the extracts of* P. nitida* fruits and *G. kola* with antibiotics did not show any case of antagonism (FIC ≥ 4) meanwhile indifference was observed in some cases of the associations of the extracts with FEP, CLX, and AMP (see Tables 5 and 6, Supplemental Material S2). Many cases of synergy were observed in most of the strains with the associations *G. kola*/ERY against CM64, *P. nitida*/NOR against KP63, and *P. nitida*/ERY against PA124.

## 4. Discussion

### 4.1. Antibacterial Activities and Chemicals Compositions of the Tested Extracts

The phytochemical studies revealed the presence of at least two classes of secondary metabolites in each of the plant extracts. Several alkaloids, flavonoids, phenols, saponins, anthocyanins, anthraquinones, sterols, tannins, and triterpenes have been found active on pathogenic microorganisms [44, 45]. Some of these compounds were found to be present in the plant species under this study, and they could contribute to the observed antimicrobial activities of some plant extracts. The results of the phytochemical test on *G. kola* are in accordance with those obtained by Onayade et al., [46, 47]. Many compounds have been isolated from *G. kola, *such as kolaflavone and 2-hydroxybiflavone [48–50] but their antimicrobials activities have not been evaluated. However, Adegboye et al. [51] reported the activity of *G. kola* on some streptomycin-sensitive Gram-positive bacteria strain. The present study therefore provides additional information on the antibacterial potential of this plant on MDR bacteria.

The previous phytochemical analyses on hexane extract from the seeds of *G. lucida *revealed several types of compounds [8, 23]. These include terpenoids, anthocyanins, flavonoids, and saponins derivatives. This report therefore agrees well with the phytochemical data being reported herein.

The results of the phytochemical analysis of the extract of fruits of *P. nitida *are similar to those obtained by Kouitcheu [52]. Several alkaloids previously isolated from this plant include akuammicine, akuammine, akuammidine, picraphylline, picraline, and pseudoakuammigine [32, 53]. Their antibacterial activities have not yet been demonstrated but many alkaloids are known to be active on Gram-negative bacteria [33]. Differences were noted in the chemical composition of the seeds and fruits of *P. nitida, *evidently explaining the differences in the antibacterial activity of the two parts of this plant. In fact, the presence of tannins in the fruits may contributes to its better activity compared to the seeds as they were reported to inactivate the microbial adhesins, enzymes, transports proteins and cellular envelop [54].

 Extracts from *C. papaya*, *C. medica, B. coriacea*,* A. cepa*, and* C. acuminata* showed weak activities against a limited number of strains. Nonetheless, the extracts from *B. coriacea *were rather reported to have good antibacterial activities. Their weak activities as observed in the present paper could therefore be due to the multidrug resistance of the studied bacteria.

### 4.2. Effects of the Association of Some Plants Extracts with Antibiotics

Three of the most active plants extracts (*G. kola, G. lucida, *and* P. nitida*) were associated with antibiotics with the aim to evaluate the possible synergistic effects of their associations. A preliminary study using *P. aeruginosa *PA124, one of the ten MDR bacteria used in this paper, was carried out with ten antibiotics (CLX, AMP, ERY, KAN, CHL, TET, FEP, STR, CIP, and NOR) to select the appropriate sub-inhibitory concentrations of the extract to be used. The results (see Supplemental Material S1) allowed the selection of *G. kola, G. lucida *and their MIC/2 and MIC/5 as the sub-inhibitory concentrations. No antagonistic effect (FIC ≥ 4) was observed between extracts and antibiotics meanwhile indifference was observed in the case of CLX, FEP, AMP, which are *β*-lactams acting on the synthesis of the bacteria cell wall [55] (Tables 5 and 6, Supplemental Material S2). Many studies demonstrated that efflux is the mechanism of resistance of bacteria for almost all antibiotic classes [56]. It is well demonstrated that the efflux pumps reduce the intracellular concentration of antibiotics and consequently their activities [57]. The MDR bacteria strains used in this paper are known for their ability to overexpress active efflux [58]. At MIC/2, synergistic effects were noted with the association of NOR, CHL, TET (on 100% the studied bacteria), ERY (on 80%), CIP (on 70%), and *P. nitida* extract meanwhile *G. kola *extract also increased the activity of NOR, TET (on 100%), ERY, and CIP (on 70%). Plant can be considered as an efflux pumps inhibitor if a synergistic effect with antibiotics is induced on more than 70% bacteria expressing active efflux pumps [6]. Therefore, the extracts from *P. nitida *and *G. kola *probably contain compounds that can acts as EPI. The results of the present paper corroborate with those of Iwu et al. [7] reporting the existence of synergy effects between *G. kola *extract and gatifloxacin (*G. kola*/gatifloxacin in the proportions of 9/1, 8/2, 7/3, and 6/4) against *Bacillus subtilis* and the proportions of *G. kola*/gatifloxacin (at 9/1, 2/8, and 1/9) against *Staphylococcus aureus.*

The overall results of the present work provide baseline information for the possible use of the studied plants and mostly *G. Lucida, G. Kola, and P. Nitida* extracts in the treatment of bacterial infections involving MDR phenotypes. In addition, the extracts of these plants could be used in association with common antibiotics to combat multidrug resistant pathogens.

## Figures and Tables

**Table 1:** Plants used in the present study and evidence of their activities.

Plant (family); and voucher number^a^	Traditional uses	Parts used	Bioactive or potentially bioactive Components	^ b^Bioactivities of crude extracts
*Allium sativum* Liliaceae; 44810/HNC	Cardiovascular diseases, intoxication, inflammations [7], fungi and parasitic infections, respiratory diseases, and asthma [8]	Bulbs	Allicine [7]	Antimicrobial: essential oil against Haemonchus contortus [8]
*Allium cepa (*Liliaceae); 034/UDS	Cardiovascular diseases, intoxication, inflammations, bacterial and fungal infections [7]	Bulbs	Sulfur component [9]	Antimicrobial: crude extract against Ec, St, and Bs [9]
*Carica papaya *(Caricaceae); 18647/SRF-CAM	Gastroenteritis, oxidative stress, intestinal worms, hepatitis, cancer, and asthma [10]	Seeds, fruits, leaf, and bark	Alkaloids, steroids, triterpenes and flavonoids [11]	Antimicrobial: seeds, fruits, and bark methanol and aqueous extract active against Sa, Ec, Pa, Pv, St, Kp, Ec, and Bs [12]
*Buchholzia coriacea *(Capparidaceae); 32124/SRF-CAM	Gastroenteritis [7]	Seeds, bark	Alkaloids, anthraquinones, tannins, cardiaques glycosides, flavonoids glycosides, saponines, steroids, steroids terpenes [13],	Antimicrobial: Seeds methanol and aqueous extract against Sa, St, Bc, Ec [13, 14]
*Citrus medica *(Rutaceae); 65106/HNC	Atheriosclerosis, influenza, infectious diseases, urinary and cholelithiasis, hypertension, dysentery, diarrhea, rheumatism, gout, worms, anemia, seasickness, pulmonary troubles, and intestinal ailments [15]	Fruits	flavonoids, phenolics, glycosides, and steroids [16]	Antimicrobial: fruit extract against Ca, Ck, Tr, Pa, Sf, St, Ec, Sa, Kp, Pv, Bc, Bm, Bs, Bst, Cf, Mm, Pm, Shf, Stm, Sp, and Ng [16]
*Cola acuminata *(Sterculiaceae); 1729/SRFK	Cellulite, Asthenia, sexual Asthenia, physical and intellectual fatigue, and gastrointestinal infections [17]	Seeds	Alkaloids (colanine or catechin-caffeine, caffeine, kolatine) [17]	—
*Garcinia kola *(Clusiaceae) 27839/SRF-CAM	Nervous alertness and induction of insomnia, purgative, wound healing, and cancers [18, 19]	Roots, seeds, and latex	kolanone, kolaflavanone, and garciniaflavanone [20, 21]	Antimicrobial: seeds ethanol extract against Sa, Sp, Spn, and Hi [22]; cytotoxicity of fruits crude methanol extract: weak activity on leukemia CCRF-CEM and pancreatic MiaPaCa-2 cell lines [19]
*Garcinia lucida *(Clusiaceae); 17974/SRF-CAM	Gastrointestinal infections, poison, and cancers [8, 19, 23]	Bark, seeds, and roots	Dihydrochelerithrine, 6-acétonyldihydrochelerithrine, and lucidamine [24]	Antimicrobial: Seeds methylene chloride extract as *β*-lactamase inhibitor [25]; cytotoxicity of fruits crude methanol extract: weak activity on leukemia CCRF-CEM and CEM/ADR5000 cells and pancreatic MiaPaCa-2 cell lines [19]
* Picralima nitida *(Apocynaceae) 1942/SRFK	Malaria and fever [26–28], diabetes, inflammation [29, 30], and* cancers *[19]	Seeds, fruits, leaf, bark, and roots	Akuammicine, akuammidine, akuammine, picracine, picraline pseudo-akuammigine [31]*;* glycosides, saponins, tannins, flavonoids, terpenoides and alkaloids [32]	Antimicrobial: fruits aqueous, methanol and dichloromethane against PF [31]; root and stem bark (aqueous and ethanol) against Sa, Pa, Ec, and Bs [32]; cytotoxicity of fruits crude methanol extract: weak activity on leukemia CCRF-CEM cell line [19]

^a^(HNC): Cameroon National Herbarium; (SRFC): Société des réserves forestières du Cameroun; (UDs): University of Dschang; Microorganisms (*Ca: Candida albicans; Ck: Candida krusei; Bc: Bacillus cereus; Bm: Bacillus megaterium; Bs: Bacillus subtilis; Bst: Bacillus stearothermophilus; Cf: Citrobacter freundii; Ec: Escherichia coli; Hi: Haemophilus influenzae; Kp: Klebsiella pneumoniae; Mm: Morganella morganii; Ng: Neisseria gonorrhoeae; Pa: Pseudomonas aeruginosa; Pf: Plasmodium falciparum; Pm: Proteus mirabilis; Pv: Proteus vulgaris; Sa: Staphylococcus aureus; Spn: Streptococcus pneumoniae; Sp: Streptococcus pneumoniae; St: Salmonella typhi; Tr: Trichophyton rubrum; Sf: Streptococcus faeealis; Shf: Shigella flexneri; Stm: Salmonella typhimurium; Sp: Streptococcus pneumonia*). ^b^Screened activity: significant (S: CMI < 100 *μ*g/mL). Moderate (M: 100 < CMI ≤ 625 *μ*g/mL). Weak (W: CMI > 625 *μ*g/mL). Q: qualitative activity based on the determination of inhibition zone [33].

**Table 2:** Bacterial strains and features.

Strains	Features	References
*Escherichia coli*		
ATCC8739 and ATCC10536	Reference strains	
AG100	Wild-type *E. coli* K-12	[31]
AG100A	AG100 *ΔacrAB*::KAN^R^	[31, 34]
AG100A_TET_	Δ*acrAB *mutant AG100, owing *acrF *gene markedly overexpressed; TET^R^	[31]
AG102	Δ*acrAB *mutant AG100	[35]
MC4100	Wild type *E. coli *	
W3110	Wild type *E. coli *	[36]
*Enterobacter aerogenes*		
ATCC13048	Reference strains	
EA-CM64	CHL^R^ resistant variant obtained from ATCC13048 over-expressing the AcrAB pump	[37]
EA3	Clinical MDR isolate; CHL^R^, NOR^R^, OFX^R^, SPX^R^, MOX^R^, CFT^R^, ATM^R^, FEP^R^	[38]
EA27	Clinical MDR isolate exhibiting energy-dependent norfloxacin and chloramphenicol efflux with KAN^R^ and AMP^R^ and NAL^R^ and STR^R^ and TET^R^	[38, 39]
EA289	KAN sensitive derivative of EA27	[40]
EA298	EA 289 *tolC::*KAN^R^	[40]
EA294	EA 289* ΔacrAB*: *::*KAN^R^	[40]
*Enterobacter cloacae*		
ECCI69	Clinical isolates	Laboratory collection of UMR-MD1, University of Marseille, France
BM47	Clinical isolates	Laboratory collection of UMR-MD1, University of Marseille, France
BM67	Clinical isolates	Laboratory collection of UMR-MD1, University of Marseille, France
*Klebsiella pneumoniae*		
ATCC12296	Reference strains	
KP55	Clinical MDR isolate, TET^R^, AMP^R^, ATM^R^, and CEF^R^	[41]
KP63	Clinical MDR isolate, TET^R^, CHL^R^, AMP^R^, and ATM^R^	[41]
K24	AcrAB-TolC	Laboratory collection of UMR-MD1, University of Marseille, France
K2	AcrAB-TolC	Laboratory collection of UMR-MD1, University of Marseille, France
*Providencia stuartii*		
NEA16	Clinical MDR isolate, AcrAB-TolC	
ATCC29914	Clinical MDR isolate, AcrAB-TolC	[42]
PS2636	Clinical MDR isolate, AcrAB-TolC	
PS299645	Clinical MDR isolate, AcrAB-TolC	
*Pseudemonas aeruginosa*		
PA 01	Reference strains	
PA 124	MDR clinical isolate	[43]

^a^AMP, ATM^R^, CEF^R^, CFT^R^, CHL^R^, FEP^R^, KAN^R^, MOX^R^, STR^R^, and TET^R^. Resistance to ampicillin, aztreonam, cephalothin, cefadroxil, chloramphenicol, cefepime, kanamycin, moxalactam, streptomycin, and tetracycline; MDR: multidrug resistant.

**Table 3:** Extraction yields, aspects, and phytochemical composition of the plant extracts.

Scientific names	Part used	Yield (%)	Physical aspect	Phytochemical composition								
				Alkaloids	Flavonoids	Phenols	Tannins	Anthraquinones	Anthocyanins	Triterpenes	Sterols	Saponins

*Picralima nitida*	Fruits	13.56	Brown paste	+	+	+	−	+	+	+	−	+
	Seeds	17.27	Brown paste	+	+	+	+	+	−	+	−	+
*Citrus medica*	Fruits	14.06	Brown paste	−	+	+	−	−	−	−	−	−
*Allium sativum*	Dry bulbs	18.99	Yellow powder	−	−	−	−	−	−	−	−	−
	Fresh bulbs	4.04	Brown powder	−	−	−	−	−	−	−	−	−
*Buchholzia coriacea*	Seeds	6.36	Brown paste	+	−	−	−	+	−	−	−	−
*Cola acuminata*	Seeds	8.81	Brown paste	+	−	+	+	+	−	+	−	+
*Garcinia kola*	Seeds	13.56	Dark brown paste	+	−	+	+	+	−	+	−	+
*Garcinia lucida*	Seeds	23.92	Brown paste	+	+	+	+	+	−	−	−	−
*Carica papaya*	Seeds	6.33	Oily paste	+	+	+	−	−	−	−	−	−
*Allium cepa*	Fresh bulbs	18.93	Brown paste	−	+	+	+	−	−	−	−	−
	Dry bulbs	49.26	Brown paste	−	+	+	−	−	−	−	−	−

(+): present; (−): absent; *The yield was calculated as the ratio of the mass of the obtained methanol extract/mass of the plant powder or fresh sample.

**Table 4:** Minimal inhibitory concentration (*μ*g/mL) of methanol extracts from the studied plants and ciprofloxacin.

Bacteria strains	Plants extracts^a^ and MIC (*μ*g/mL) in the absence and presence of PA*β*N (in bracket)												
	CAF	PNF	ASB1	ASB2	BCF	PNS	CMF	GKS	GLS	CPS	ACB1	ACB2	CIP

*E. coli*													
ATCC8739	—	1024	—	—	—	—	—	512	512	—	—	—	<0.5
ATCC10536	—	1024	—	—	—	1024	—	512	512	1024	—	—	64
W3110	1024 (1024)	512 (512)	—	—	—	— (512)	— (1024)	512 (256)	256 (128)	—	—	— (1024)	<0.5 (<0.5)
MC4100	—	512	—	—	—	1024	—	512	256	—	1024	1024	32
AG100A	1024	512 (128)	—	— (512)	—	— (1024)	—	1024 (1024)	256 (64)	1024 (1024)	—	—	16 (8)
AG100Atet	1024 (1024)	1024 (512)	—	—	—	—	—	256 (256)	512 (512)	1024 (1024)	1024 (1024)	—	32 (8)
AG102	—	512 (128)	—	— (1024)	— (1024)	— (1024)	— (1024)	256 (64)	512 (256)	512 (512)	—	—	32 (16)
AG100	—	512	—	—	1024	—	—	256	256	1024	—	1024	0.5
*E. aerogenes*													
ATCC13048	—	512	—	—	—	—	—	512	256	—	—	—	1
EA294	—	1024	—	—	—	—	—	512	256	—	1024	—	64
CM64	1024	512	—	—	1024	—	1024	256	256	—	512	—	32
EA3	—	512	—	—	—	—	—	512	256	—	—	—	32
EA298	—	— (512)	—	—	—	—	—	512 (128)	256 (128)	—	—	—	1 (<0.5)
EA27	1024 (1024)	512 (512)	—	—	—	—	—	256 (256)	256 (256)	1024 (1024)	—	—	1 (<0.5)
EA289	—	1024 (1024)	—	—	—	—	— (1024)	512 (512)	512 (256)	—	—	—	64 (32)
*K. pneumoniae*													
ATCC11296	1024 (1024)	512 (256)	—	— (1024)	— (1024)	—	—	512 (512)	256 (128)	— (512)	—	—	<0.5 (<0.5)
KP55	512 (512)	512 (256)	—	—	—	—	—	512 (512)	128 (128)	1024 (1024)	1024 (1024)	— (1024)	32 (4)
KP63	—	512	—	—	—	—	—	512	512	—	—	—	32
K2	—	1024	—	—	—	—	—	512 (256)	256 (128)	1024 (1024)	— (512)	—	32 (8)
K24	512	512	—	—	—	—	—	512	256	1024	1024	—	32
*P. aeruginosa*													
PA01	—	1024 (1024)	—	—	—	—	—	512 (512)	512 (512)	—	—	—	32 (4)
PA124	—	512	—	—	—	—	—	1024	256	1024	—	—	128
*P. stuartii*													
ATCC29916	1024 (1024)	1024 (1024)	—	—	—	—	—	512 (512)	256 (128)	1024 (1024)	—	—	>64 (16)
NAE16	1024	512	—	—	—	1024	—	256	256	1024	1024	—	64
PS2636	—	1024	—	—	—	—	—	128	128	—	—	—	64
PS299645	1024 (1024)	1024 (1024)	—		1024 (1024)	1024 (1024)	1024 (1024)	256 (256)	128 (128)	1024 (1024)	—	—	<0.5 (<0.5)
*E. cloacae*													
BM47	—	256	—	—	—	—	—	256	256	—	—	1024	64
ECCI69	1024	512	—	—	1024	—	1024	128	128	—	—	—	128
BM67	1024	512	—	—	—	—	—	256	256	1024	1024	—	32

(—) MIC greater than 1024 *μ*g/mL; ^a^Extract from CAF: *Cola acuminata* fruit; PNF: *Picralima nitida* fruits; ASB1: *Allium sativum* dry bulbs; ASB2: *Allium sativum* fresh bulbs; BCF: *Buchholsia coriacea* fruits; PNS: *Picralima nitida *seeds; CMF: *Citrus médica* fruits juice; GKS: *Garcinia kola* seeds; GLS: *Garcinia lucida* seeds; CPS: *Carica papaya seeds*; ACB1: *Allium cepa* fresh bulbs; ACB2: *Allium cepa* dry bulbs; CIP: ciprofloxacin.

**Table 5:** MIC of different antibiotics after the association of the extract of *Picralima nitida* fruits at MIC/2, MIC/5 against ten MDR bacteria strains.

Antibiotics	Extract concentration	Bacterial strains, MIC (*μ*g/mL) of antibiotics in the absence and presence of the extract									
		AG100	AG100Atet	AG102	CM64	EA3	EA27	EA289	KP55	KP63	PA124

CIP	0 MIC/2 MIC/5	≤0.5 ≤0.5 ≤0.5	128 16(8)^S^ 32(4)^S^	32 16(2)^S^ 16(2)^S^	≤0.5 ≤0.5 ≤0.5	256 64(4)^S^ 128(2)^S^	≤0.5 ≤0.5 ≤0.5	64 16(4)^S^ 32(2)^S^	256 128(2)^S^ 256(1)^I^	128 64(2)^S^ 64(2)^S^	32 8(4)^S^ 8(4)^S^
CHL	0 MIC/2 MIC/5	4 2(2)^S^ 4(1)^I^	>512 64(>8)^S^ 128(>4)^S^	128 16(8)^S^ 32(4)^S^	512 64(8)^S^ 128(4)^S^	512 64(8)^S^ 128(4)^S^	64 8(8)^S^ 32(2)^S^	512 64(8)^S^ 128(4)^S^	32 16(2)^S^ 16(2)^S^	512 128(4)^S^ 256(2)^S^	64 8(8)^S^ 32(2)^S^
STR	0 MIC/2 MIC/5	4 2(2)^S^ 2(2)^S^	>512 256(>2)^S^ 512(>1)	≤0.5 ≤0.5 ≤0.5	512 256(2)^S^ 512(1)^I^	>512 64(8)^S^ 256(>2)^S^	16 16(1)^I^ 16(1)^I^	16 16(1)^I^ 16(1)^I^	16 16(1)^I^ 16(1)^I^	128 128(1)^I^ 128(1)^I^	>512 128 512
AMP	0 MIC/2 MIC/5	32 16(2)^S^ 16(2)^S^	>512 512(1)^I^ >512	256 64(4)^S^ 64(4)^S^	512 512(1)^I^ 512(1)^I^	>512 128(>4)^S^ 512(>1)	64 64(1)^I^ 64(1)^I^	>512 >512 >512	>512 >512 >512	>512 >512 >512	>512 16(>32)^S^ 16(>32)^S^
TET	0 MIC/2 MIC/5	64 16(4)^S^ 32(2)^S^	256 128(2)^S^ 256(1)^I^	8 1(8)^S^ 4(2)^S^	128 32(4)^S^ 64(2)^S^	512 64(8)^S^ 128(4)^S^	8 2(4)^S^ 4(2)^S^	32 8(4)^S^ 8(4)^S^	8 4(2)^S^ 4(2)^S^	16 8(2)^S^ 8(2)^S^	8 2(4)^S^ 4(2)^S^
CLX	0 MIC/2 MIC/5	64 32(2)^S^ 32(2)^S^	>512 >512 >512	>512 128(>4)^S^ 256(>2)^S^	>512 256 >512	>512 >512 >512	>512 >512 >512	>512 >512 >512	>512 >512 >512	>512 >512 >512	>512 512(>1) >512
KAN	0 MIC/2 MIC/5	≤4 ≤4 ≤4	512 128(4)^S^ 128(4)	16 16(1)^I^ 16(1)^I^	≤4 ≤4 ≤4	≤4 ≤4 ≤4	>512 512(>2)^S^ >512	32 ≤4(>8)^S^ 16(2)^I^	32 8(4)^S^ 8(4)^S^	512 128(4)^S^ 512(1)^I^	128 64(2)^S^ 64(2)^S^
ERY	0 MIC/2 MIC/5	64 32(2)^S^ 64(1)^I^	512 256(2)^S^ 256(2)^S^	16 16(1)^I^ 16(1)^I^	256 32(8)^S^ 256(1)^I^	32 8(4)^S^ 16(2)^S^	8 4(2)^S^ 8(1)^I^	128 128(1)^I^ 128(1)^I^	64 16(4)^S^ 32(2)^S^	128 64(2)^S^ 128(1)^I^	128 8(16)^S^ 8(16)^S^
NOR	0 MIC/2 MIC/5	32 16(2)^S^ 32(1)^I^	512 128(4)^S^ 128(4)^S^	128 32(4)^S^ 64(2)^S^	16 8(2)^S^ 16(1)^I^	16 4(4)^S^ 16(1)^I^	32 16(2)^S^ 16(2)^S^	64 32(2)^S^ 32(2)^S^	64 32(2)^S^ 32(2)^S^	64 4(8)^S^ 16(4)^S^	128 32(4)^S^ 32(4)^S^
FEP	0 MIC/2 MIC/5	512 256(2)^S^ 512(1)^I^	512 128(4)^S^ 512(1)^I^	512 512(1)^I^ 512(1)^I^	>512 256(>4)^S^ >512	256 64(4)^S^ 128(2)^S^	512 252(2)^S^ 512(1)^I^	512 512(1)^I^ 512(1)^I^	>512 >512 >512	512 256(2)^S^ 512(1)^I^	512 512(1)^I^ 512(1)^I^

(): fold increase in MIC values of the antibiotics after association with plants extract; S: synergy, I: indifference; AMP: ampicillin; FEP: cefepime; CHL: chloramphenicol; KAN: kanamycin; NOR: norfloxacin; STR: streptomycin; TET: tetracycline; CIP: ciprofloxacin; CLX: cloxacillin; ERY: erythromycin.

**Table 6:** MIC of different antibiotics after the association of the extract of *Garcinia kola* seeds at MIC/2, MIC/5 against ten MDR bacteria strains.

Antibiotics	Extract concentration	Bacterial strains, MIC (*μ*g/mL) of antibiotics in the absence and presence of the extract									
		AG100	AG100Atet	AG102	CM64	EA3	EA27	KP55	KP63	EA289	PA124

CIP	0 MIC/2 MIC/5	≤0.5 ≤0.5 ≤0.5	128 64(2)^S^ 64(2)^S^	32 8(4)^S^ 8(4)^S^	≤0.5 ≤0.5 ≤0.5	256 128(2)^S^ 128(2)^S^	≤0.5 ≤0.5 ≤0.5	256 128(2)^S^ 256(1)^I^	128 32(4)^S^ 128(1)^I^	64 32(2)^S^ 64(1)^I^	32 <0.5 16(2)^S^
CHL	0 MIC/2 MIC/5	4 4(1)^I^ 4(1)^I^	>512 512(>1) 512(>1)	128 16(8)^S^ 32(4)^S^	512 256(2)^S^ 512(1)^I^	512 512(1)^I^ 512(1)^I^	64 8(8)^S^ 16(4)^S^	32 32(1)^I^ 32(1)^I^	512 128(4)^S^ 256(2)^S^	512 128(4)^S^ 256(2)^S^	64 32(2)^S^ 32(2)^ S^
STR	0 MIC/2 MIC/5	4 2(2)^S^ 2(2)^S^	>512 >512 >512	<0.5 <0.5 <0.5	512 256(2)^S^ 512(1)^I^	>512 >512 >512	16 8(2)^S^ 16(1)^I^	16 8(2)^S^ 16(1)^I^	128 128(1)^I^ 128(1)^I^	16 8(2)^S^ 16(1)^I^	>512 16(>32)^S^ 128(>4)^S^
AMP	0 MIC/2 MIC/5	32 8(4)^S^ 32(1)^I^	>512 >512 >512	256 128(2)^S^ 128(2)^S^	>512 >512 >512	>512 >512 >512	64 64(1)^I^ 64(1)^I^	>512 >512 >512	>512 512 512	>512 >512 >512	>512 64(>8)^S^ 256(>2)^S^
TET	0 MIC/2 MIC/5	64 32(2)^S^ 32(2)^S^	256 128(2)^S^ 128(2)^S^	8 2(4)^S^ 2(4)^S^	128 64(2)^S^ 64(2)^S^	512 256(2)^S^ 256(2)^S^	8 2(4)^S^ 4(2)^S^	8 2(4)^S^ 4(2)^S^	16 4(4)^S^ 16(1)^I^	32 8(4)^S^ 8(4)^S^	8 4(2)^S^ 8(1)^I^
CLX	0 MIC/2 MIC/5	32 16(2)^S^ 32(1)^I^	>512 >512 >512	>512 512 512	>512 >512 >512	>512 >512 >512	128 32(4)^S^ 128(1)^I^	>512 >512 >512	>512 >512 >512	>512 >512 >512	>512 128(>4)^S^ >512
KAN	0 MIC/2 MIC/5	≤4 ≤4 ≤4	512 32(16)^S^ 256(2)^S^	16 16(1)^I^ 16(1)^I^	≤4 ≤4 ≤4	≤4 ≤4 ≤4	>512 512 >512	32 4(8)^S^ 16(2)^S^	512 256(2)^S^ 512(1)^I^	32 ≤4(>8)^S^ 32(1)^ S^	128 16(8)^S^ 64(2)^S^
ERY	0 MIC/2 MIC/5	64 16(4)^S^ 64(1)^I^	512 32(16)^S^ 512(1)^I^	16 16(1)^I^ 16(1)^I^	256 16(16)^S^ 32(8)^S^	64 16(4)^S^ 16(4)^S^	8 4(2)^S^ 8(1)^I^	64 64(1)^I^ 64(1)^I^	128 16(8)^S^ 32(4)^S^	128 128(1)^I^ 128(1)^I^	256 32(8)^S^ 256(1)^I^
NOR	0 MIC/2 MIC/5	32 8(4)^S^ 16(2)^S^	512 128(4)^S^ 256(2)^S^	128 64(2)^S^ 128(1)^I^	16 4(4)^S^ 8(2)^S^	16 8(2)^S^ 8(2)^S^	32 8(4)^S^ 32(1)^I^	128 32(4)^S^ 128(1)^I^	64 8(8)^S^ 16(4)^S^	64 32(2)^S^ 32(2)^S^	256 256(1)^I^ 256(1)^I^
FEP	0 MIC/2 MIC/5	512 512(1)^I^ 512(1)^I^	>512 512 (>1) 512(>1)	>512 >512 >512	>512 256(>2)^S^ 256(>2)^S^	>512 >512 >512	>512 >512 >512	>512 >512 >512	512 256(2)^S^ 512(1)^S^	512 512(1)^I^ 512(1)^I^	>512 512(>1) 512(>1)

(): fold increase in MIC values of the antibiotics after association with plants extract; S: synergy; I: indifference; AMP: ampicillin; FEP: cefepime; CHL: chloramphenicol; KAN: kanamycin; NOR: norfloxacin; STR: streptomycin; TET: tetracycline; CIP: ciprofloxacin; CLX: cloxacillin; ERY: erythromycin.
